# Retinal Microvasculature and Choriocapillaris Flow Deficit in Relation to Serum Uric Acid Using Swept-Source Optical Coherence Tomography Angiography

**DOI:** 10.1167/tvst.11.8.9

**Published:** 2022-08-10

**Authors:** Yu Lu, Jing Yue, Jian Chen, Xue Li, Lanhua Wang, Wenyong Huang, Jianyu Zhang, Ting Li

**Affiliations:** 1 Department of Rheumatology, The Third Affiliated Hospital of Guangzhou Medical University, Guangzhou, Guangdong, China; 2 State Key Laboratory of Ophthalmology, Zhongshan Ophthalmic Center, Sun Yat-Sen University, Guangzhou, China

**Keywords:** uric acid, choriocapillaris flow, SS-OCTA, retinal vessel density, Chinese

## Abstract

**Purpose:**

To explore the relationship between serum uric acid (SUA) and retinochoroidal microcirculation in the Chinese population.

**Methods:**

This prospective cross-sectional study was conducted among the residents of Guangzhou, southern China. A commercially available optical coherence tomography angiography (OCTA) device was used to obtain the superficial vessel density (SVD) and deep vessel density in the retina and the choriocapillaris flow deficit (CFD) in the macular region. Univariable and multivariable linear regression models were used to assess the association of hyperuricemia and SUA levels with OCTA parameters.

**Results:**

A total of 638 participants with normal SUA and 296 participants with hyperuricemia were included in the study. Parafoveal SVD was significantly reduced among the participants with hyperuricemia compared to participants with normal SUA (*P* < 0.001), while the parafoveal CFD was higher in hyperuricemic participants than those of normal SUA levels (*P* = 0.007). After adjusting for potential confounders, greater SUA levels was associated with lower SVD (β = −0.078; *P* < 0.001) and greater CFD (β = 0.015; *P* = 0.011). Gender difference analysis indicated that a 10-µmol/L increase in SUA levels among the female participants led to a 0.144 decrease in SVD (*P* < 0.001), but it was not statistically significant for the male participants (*P* = 0.653).

**Conclusions:**

An elevated uric acid level and its fluctuations were independently associated with impaired retinal and choroidal microcirculation using OCTA in the study population. Women appear to be more sensitive to high SUA levels than men.

**Translational Relevance:**

Elevating uric acid concentration may play a role in the development and progression of cardiovascular diseases through microvascular alteration, as demonstrated by OCTA parameters.

## Introduction

The retina provides a window for direct evaluation of early structural alterations of the vascular system in vivo.^1^^,^^2^ Alterations in the retinal and choroidal vessels are significantly associated with cardiovascular diseases such as myocardial infarction and stroke and ocular diseases such as glaucoma and diabetes retinopathy.^3^^–^^5^ Retinal and choroidal vasculature evaluation is traditionally performed using fluorescence angiography and indocyanine green angiography, which require intravenous dye injection and pose a risk of fatal complications. In addition, the retinal vessel plexus and the choriocapillaris (CC) cannot be effectively differentiated with this method because of their limited resolution and two-dimensional images.^6^

Optical coherence tomography angiography (OCTA) is a new noninvasive technique that enables accurate quantification of the retinal vasculature and the CC in vivo.^7^ Recent studies have demonstrated that OCTA performs excellently in intragrader reproducibility and intergrader agreement.^8^^,^^9^ By using OCTA, it has been reported that superficial retinal vessel density is significantly associated with sex, while deep retinal vessel density is influenced by serum creatine and axial length.^10^ Furthermore, choriocapillaris flow deficit (CFD) is related to age, blood pressure, and serum lipid levels.^11^

Uric acid (UA) is considered a significant mediator of the vascular system.^12^^–^^14^ It has been reported that hyperuricemia can predict the progression of hypertension, chronic kidney disease, and diabetic vascular complications.^15^^–^^20^ However, other studies have found no significant association between serum UA (SUA) and diabetic vascular complications.^21^^,^^22^ To our knowledge, no study has analyzed the association between SUA and retinal and choroidal blood flow by using OCTA. Therefore, the relationship between SUA and ocular microcirculation remains unclear. To fill the knowledge gap, this OCTA study was conducted to explore the associations of SUA level with the retinal microvasculature of and CC flow in the Chinese population.

## Methods

### Study Population

This prospective cross-sectional study was conducted at the Preventive Ophthalmology Department of Zhongshan Ophthalmic Centre, Sun Yat-sen University, Guangzhou. All participants provided written informed consent before participating in this study. The study protocol was approved by the Ethics Committee of the Zhongshan Ophthalmic Centre (2017KYPJ094), and the study was conducted in accordance with the tenets of the Declaration of Helsinki.

Individuals aged 30 to 80 years and with refractive error less than ±5 diopters (D), an axial length between 21 and 25 mm, and intraocular pressure (IOP) less than 21 mmHg, without a history of ocular diseases, were recruited for this study. Subjects with any of the following conditions were excluded from the analysis: evidence of optic nerve or retinal disease (e.g., glaucoma or diabetic retinopathy), systemic lesions that may compromise the visual system, ocular trauma, history of laser treatment, lesions affecting the optic nerve and retina, eyes with corneal ulcers, severe lens opacity, pterygium, and a history of severe systemic diseases, such as ischemic heart disease, malignancy, and stroke. Subjects with inadequate fundus or OCTA image quality were also excluded. If both eyes were eligible, only the right eye was adopted to avoid potential bias between the eyes of the same subject. If a subject had only one eligible eye, the eligible eye was selected for analysis.

### Systemic and Ocular Examinations

Each participant underwent comprehensive physical and ocular examinations. Fasting blood samples were used to measure hemoglobin A1c (HbA1c), SUA (or UA), serum creatinine, total cholesterol, triglyceride, low-density lipoprotein cholesterol (LDL-C), high-density lipoprotein cholesterol (HDL-C), and C-reactive protein. Clean midstream urine samples were used to quantify urine microalbumin. Hyperuricemia was defined as an SUA level >420 µmol/L in men and >360 µmol/L in women. Height, weight, waist, hip, systolic blood pressure (SBP), and diastolic blood pressure (DBP) were also measured using standardized protocols. Body mass index (BMI = weight [kg]/height [m^2^]) and mean arterial pressure ([SBP + 2 × DBP]/3) were calculated. In-person interviews by trained interviewers were performed to collect information on detailed ophthalmic and systemic medical history by using a standard questionnaire.

The ophthalmic examinations included slit-lamp biomicroscopy, visual acuity, IOP, refraction, ocular biometry measurements, optical coherence tomography (OCT), and OCTA. Visual acuity was measured using the Early Treatment Diabetic Retinopathy Study (ETDRS) LogMAR E chart (Precision Vision, Villa Park, IL, USA). Refractive error was measured by an automated refractometer (KR8800; Topcon, Tokyo, Japan), and IOP was measured by a noncontact IOP meter (Topcon CT-80A; Topcon). Central corneal thickness, lens thickness, axial length, and anterior chamber depth were measured by an optical low-coherence reflectometer (Lenstar LS900; Haag-Streit AG, Koeniz, Switzerland). After pupil dilatation, two 45° fundus color photographs were obtained using a digital fundus camera (Canon CR-2, Tokyo, Japan) centered on the macula and the optic disc, respectively.

### Swept-Source OCT and Swept-Source OCTA Imaging

All operations were performed by the same experienced technician. All subjects were confirmed to have no history of caffeine or alcohol intake for at least 24 hours before imaging. Macular structure and blood flow imaging were performed using a commercially available swept-source (SS)–OCT/A device (DRI Triton; Topcon), which employs a tunable laser using 1050 nm as a light source with a high-speed sweep (100,000 A-scans/s) and high resolution (8-µm axial resolution, 20-µm transverse resolution of 20 µm). A 7 × 7-mm^2^ volume scan protocol centering on the fovea was used to obtain structural images of the macula, and the retinal and choroidal thicknesses were automatically calculated using the built-in software (IMAGEnet 6, version 1.22, NIH, Bethesda, MD, USA). Two trained technicians reviewed the retina–choroid and choroid–sclera boundaries in all the images, and manual adjustments were made if error segmentation existed. Images with artifacts and uncorrected segmentation errors were excluded.

En face OCTA images were obtained using a 6 × 6-mm Angio protocol centering on the macula, with a scan density of 320 A-scans × 320 B-scans. Each raster scan consisted 320 B-scans, and each B-scan position was repeatedly scanned four times.^23^ The motion and projection artifacts were reduced by using the built-in eye-tracking system and artifact elimination algorithm during imaging.^24^ Automatic segmentation was performed by the built-in IMAGEnet 6.0 (version 1.22) to obtain en face images of the superficial capillary plexus, the deep capillary plexus, and the CC layers.^25^ The superficial capillary plexus was delineated by 2.6 µm below the internal limiting membrane to 15.6 µm below the junction between the inner plexiform and the inner nuclear layers, the deep capillary plexus was delineated by 15.6 µm below the inner plexiform, and CC was defined as from the Bruch membrane to 10.4 µm below the Bruch membrane. The reliability of the automatic layer boundaries was manually checked and corrected for images with wrong outlined layer boundaries. Images with image quality score (IQS) <60 (range, 0–100), motion artifacts, segmentation errors, missing signal, or decentration were excluded for further analysis.

**Figure 1.**
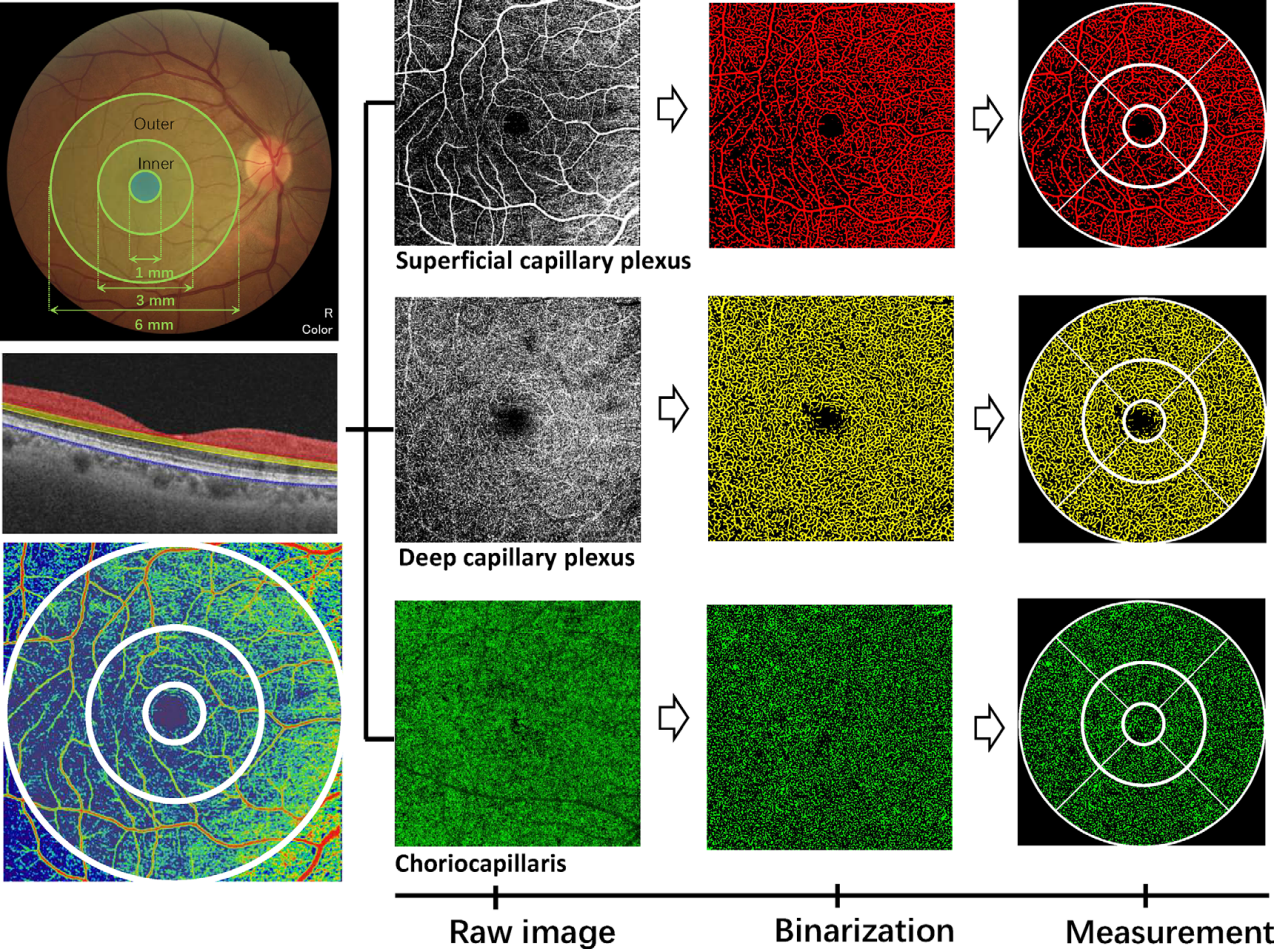
The swept-source optical coherence tomographic angiography provides information of macular capillary plexus in the ETDRS circles of the superficial and deep layer, as well as the underneath choriocapillaris layer. *Left top*: fundus color photographs and corresponding ETDRS scan areas. *Left middle*: OCTA B-scan with color-coded angiography segmented in three vascular plexuses: superficial capillary network in *red*, deep capillary network in *yellow*, and choriocapillaris in *blue*. *Left bottom*: superficial retinal blood flow en face maps. *First row*: the OCTA scans of the superficial capillary network. *Second row*: the OCTA scans of the deep capillary network. *Third row*: the OCTA scans of the choriocapillaris.

The Fiji software (version 2.0; National Institutes of Health, Bethesda, MD, USA) was used to quantify the superficial vessel density (SVD), deep vessel density (DVD), and CFD in the parafoveal region according to methods published in previous literature (Fig. 1).^24^^,^^26^ The magnification effects due to axial length (AL) were corrected using the Littman and Bennett formulas.^27^ The macula was divided into nine subregions according to ETDRS grids, consisting of three rings of 1.0 mm, 3.0 mm, and 6 mm. Parafoveal SVD and DVD were defined as the vascular density in the inner and outer ring regions. For CFD quantification, binarization was performed using the Phansalkar method, with a window radius of 17.58 µm (one to two intercapillary distances for window diameter). The CFD was calculated from the ratio of the total area of flow voids to the total area, excluding the area of projection artifacts.^11^

### Statistical Analysis

All statistical analyses were performed using STATA version 16.0 (StataCorp, College Station, TX, USA). The skewness–kurtosis test and histogram were used to test the normality of variables. Characteristics between the groups were compared using Student's *t*-test for normal distributions, rank-sum test for skewed distributions, or χ^2^ test for categorical data where appropriate. Univariable and multivariable linear regression analyses were used to estimate the potential effect of SUA on retinal vessel density and CFD in the parafoveal region. For multivariate regression, the following variables were adjusted: age, sex, BMI, waist-to-hip ratio (WHR), axial length, total cholesterol, triglyceride, HDL-C, LDL-C, HbA1c, estimated glomerular filtration ratio, microalbuminuria, and diagnosis of diabetes mellitus. All *P* values presented were two-sided, and *P* < 0.05 was regarded as statistically significant.

## Results

### Characteristics of the Study Participants

Table 1 presents the demographic and clinical characteristics of the study participants. A total of 638 participants (638 eyes) with normal SUA levels and 296 participants (296 eyes) with hyperuricemia were included in the study. The subjects with hyperuricemia tended to be older and had higher BMI and WHR, higher blood pressure, more commodities with diabetes, higher cholesterol, higher triglycerides, lower HDL-C, greater urine microalbumin and C-reactive protein, thinner ganglion cell–inner plexiform layer thickness, thinner retinal thickness, and lower IQS of OCTA imaging (all *P* < 0.05). Participants with and without hyperuricemia were similar in sex, HbA1c, IOP, central corneal thickness, anterior chamber depth, lens thickness, and axial length (all *P* > 0.05).

**Table 1.** Demographic and Clinical Characteristics of the Study Subjects

Characteristic	Normal SUA	Hyperuricemia	*P* Value^a^
No. of subjects (eyes)	638 (638)	296 (296)	—
Male, *n* (%)	176 (30.0)	120 (34.5)	0.158^b^
Age, y	59.5 ± 9.1	62.1 ± 8.8	<0.001
BMI, kg/m^2^	23.4 ± 3.1	24.5 ± 2.9	<0.001
WHR	0.88 ± 0.06	0.90 ± 0.06	<0.001^c^
SBP, mm Hg	125.2 ± 18.0	131.4 ± 16.6	<0.001
DBP, mm Hg	68.6 ± 10.3	70.4 ± 10.0	0.014
Diagnosis of diabetes, *n* (%)	157 (28.9)	139 (35.6)	0.028^b^
HbA1c, %	6.35 ± 1.26	6.39 ± 1.04	0.605
Triglycerides, mmol/L	2.12 ± 1.58	2.77 ± 1.82	<0.001
Total cholesterol, mmol/L	5.06 ± 1.00	5.22 ± 1.15	0.030
LDL-C, mmol/L	3.15 ± 0.88	3.22 ± 0.98	0.320
HDL-C, mmol/L	1.37 ± 0.41	1.22 ± 0.39	<0.001
SUA, µmol/L	311.7 ± 51.0	443.5 ± 70.1	<0.001
Serum creatinine, µmol/L	68.3 ± 15.2	73.5 ± 20.6	<0.001
Microalbuminuria, mg/mL	1.38 ± 3.94	2.71 ± 8.73	0.002^c^
C-reactive protein, mg/L	1.79 ± 3.26	2.27 ± 2.91	0.031^c^
BCVA, ETDRS letters	82.5 ± 4.2	81.9 ± 4.9	0.055
IOP, mmH g	15.8 ± 2.4	15.9 ± 2.4	0.417
Central corneal thickness, µm	539.1 ± 31.2	541.1 ± 33.0	0.373
Anterior chamber depth, mm	2.5 ± 0.4	2.5 ± 0.4	0.301
Lens thickness, mm	4.6 ± 0.4	4.6 ± 0.3	0.184
AL, mm	23.5 ± 0.9	23.4 ± 0.9	0.670
Average GCIPL thickness, µm	71.3 ± 5.3	70.4 ± 5.3	0.010
Average retinal thickness, µm	273.8 ± 22.5	269.5 ± 25.4	0.017
IQS	72.0 ± 6.8	70.8 ± 7.4	0.017

All data presented as mean ± standard deviation unless otherwise indicated. BCVA, best-corrected visual acuity; GCIPL, ganglion cell–inner plexiform layer.

a Comparison was conducted between participants with and without hyperuricemia using Student's *t*-test unless otherwise indicated.

b χ^2^ test.

c Rank-sum test.

### Comparisons of OCTA Parameters Between the Groups

**Figure 2.**
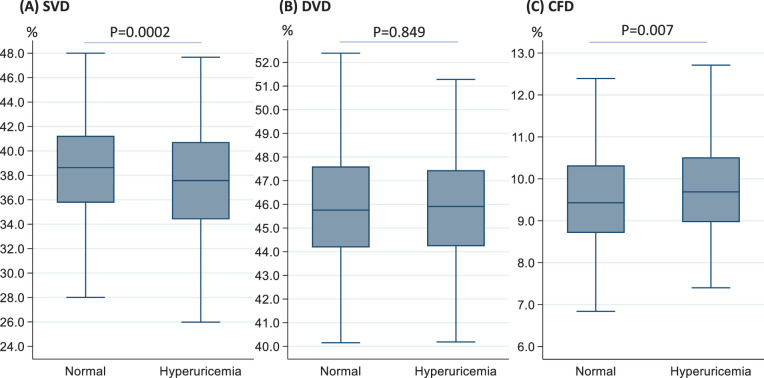
Distribution of retinal vessel density and choriocapillaris flow deficit between the participants with and without hyperuricemia. Student's *t*-test for statistical significance.

**Figure 3.**
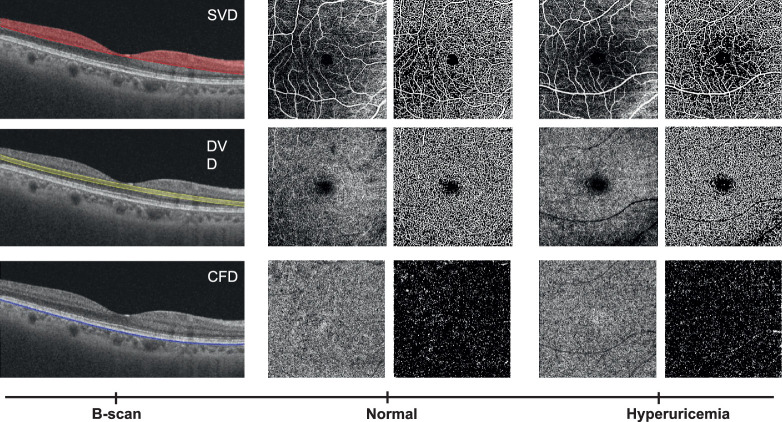
Representative examples of optical coherence tomographic angiography measurements in normal participants and in patients with hyperuricemia. *First row*: the OCTA scans of the superficial capillary network. *Second row*: the OCTA scans of the deep capillary network. *Third row*: the OCTA scans of the choriocapillaris in normal participants and in patients with hyperuricemia. *Left column*: corresponding OCTA B-scan with color-coded angiography segmented in three vascular plexuses: superficial capillary network in *red*, deep capillary network in *yellow*, and choriocapillaris in *blue*.

Table 2 shows the comparisons of the average vessel density and flow deficit in the parafoveal regions between the two groups. Parafoveal SVD (37.40 ± 4.19 vs. 38.45 ± 3.87, *P* < 0.001) was significantly reduced among participants with hyperuricemia compared to participants with normal SUA levels, while levels of parafoveal CFD were higher among participants with hyperuricemia compared to participants with normal SUA levels (9.80 ± 1.31 vs. 9.56 ± 1.28, *P* = 0.007). There were no significant differences in the levels of parafoveal DVD (*P* = 0.849) between groups (Fig. 2). When stratified by sex, only females had lower SVD and higher CFD in the parafoveal region (Table 2). Both groups had similar parafoveal DVDs in overall and subgroup analyses (all *P* > 0.05). Figure 3 shows representative examples of OCTA measurements in normal participants and in patients with hyperuricemia. Additionally, regional topographical distributions of OCTA parameters in both groups are presented in Supplementary Table S1.

**Table 2.** Comparisons of Retinal Microvasculature and Choriocapillaris Flow in Parafoveal Region Between the Subjects With Normal SUA and Hyperuricemia

Characteristic	Normal SUA, Mean ± SD	Hyperuricemia, Mean ± SD	*P* Value^a^
Parafoveal SVD, %			
All	38.45 ± 3.87	37.40 ± 4.19	<0.001
Female	38.60 ± 3.66	37.05 ± 4.20	<0.001
Male	38.19 ± 4.21	37.91 ± 4.14	0.553
Parafoveal DVD, %			
All	45.88 ± 2.40	45.91 ± 2.42	0.849
Female	45.99 ± 2.32	45.86 ± 2.62	0.541
Male	45.68 ± 2.53	45.99 ± 2.10	0.249
Parafoveal CFD, %			
All	9.56 ± 1.28	9.80 ± 1.31	0.007
Female	9.34 ± 1.19	9.58 ± 1.16	0.028
Male	9.95 ± 1.34	10.14 ± 1.44	0.236

a Student's *t*-test.

### Associations of SUA Levels with OCTA Parameters

**Figure 4.**
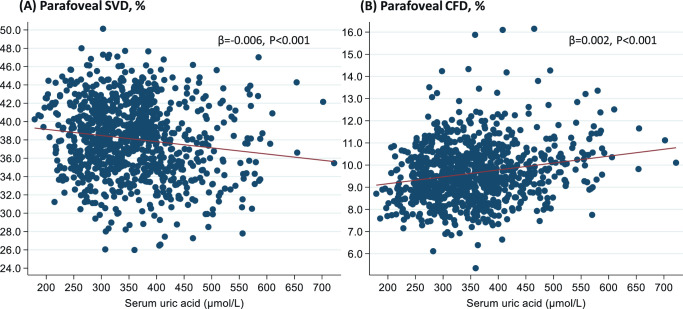
Scattering plots showing the associations of serum uric acid and parafoveal superficial vessel density and choriocapillaris flow deficit.

Table 3 summarizes the relationship between SUA level and OCTA parameters. In the univariable linear regression analysis, increased SUA levels were associated with lower SVD (−0.067; 95% confidence interval [CI], −0.098 to −0.037; *P* < 0.001) and higher CFD (0.031; 95% CI, 0.021 to 0.041; *P* < 0.001) in all participants (Fig. 4). After adjusting age, sex, BMI, WHR, axial length, total cholesterol, triglyceride, HDL-C, LDL-C, HbA1c, estimated glomerular filtration ratio, microalbuminuria, and diagnosis of diabetes mellitus, great SUA level remained significantly associated with reduced SVD (−0.078; 95% CI, −0.116 to −0.039; *P* < 0.001) and elevated CFD (0.015; 95% CI, 0.003 to 0.027; *P* = 0.011). No significant association between DVD and SUA levels was found. Further subgroup analysis stratified by gender showed that SUA levels were negatively associated with SVD only among females (−0.144; 95% CI, −0.194 to −0.094; *P* < 0.001), while higher SUA levels were associated with greater CFD in both males (0.018; 95% CI, 0.002 to 0.033; *P* = 0.024) and females (0.021; 95% CI, 0.001 to 0.041; *P* = 0.040) in a multivariable model.

**Table 3.** Univariable and Multivariable Analyses of the Associations of SUA Levels (Per 10-µmol/L Increase) With the Retinal Vessel Density and Choriocapillaris Flow Deficit

	Univariable Model		Multivariable Model^a^	
Characteristic	β (95% CI)	*P* Value	β (95% CI)	*P* Value
SVD				
All	−0.067 (−0.098 to −0.037)	<0.001	−0.078 (−0.116 to −0.039)	<0.001
Female	−0.163 (−0.208 to −0.119)	<0.001	−0.144 (−0.194 to −0.094)	<0.001
Male	0.002 (−0.048 to 0.052)	0.944	−0.014 (−0.075 to 0.047)	0.653
DVD				
All	−0.015 (−0.033 to 0.004)	0.116	−0.005 (−0.028 to 0.017)	0.641
Female	−0.034 (−0.063 to −0.006)	0.018	−0.017 (−0.049 to 0.015)	0.291
Male	0.009 (−0.020 to 0.037)	0.552	−0.001 (−0.036 to 0.033)	0.938
CFD				
All	0.031 (0.021 to 0.041)	<0.001	0.015 (0.003 to 0.027)	0.011
Female	0.028 (0.014 to 0.042)	<0.001	0.018 (0.002 to 0.033)	0.024
Male	0.011 (−0.005 to 0.028)	0.175	0.021 (0.001 to 0.041)	0.040

a Adjusted for age, sex, body mass index, waist-to-hip ratio, axial length, total cholesterol, triglyceride, HDL-C, LDL-C, HbA1c, estimated glomerular filtration ratio, microalbuminuria, and diagnosis of diabetes mellitus.

## Discussion

OCTA is widely used in clinical research and practice, with the advantages of being noninvasive, fast scanning, and high resolution. Although several studies have reported the distributions of retinal vessel densities and CFD in healthy volunteers, the effects of SUA on OCTA parameters remain unclear. This study demonstrated a significant association of SUA with chorioretinal microcirculation independently of other traditional cardiovascular risk factors in this Chinese population. A higher level of SUA was related to lower SVD, and this association was more pronounced in females. Furthermore, SUA was positively associated with CFD in the multivariable analysis. To the best of our knowledge, this is the first study to evaluate the effects of SUA variations on chorioretinal microvasculature on OCTA.

We observed a dose–response association of higher SUA levels with lower SVD even after adjusting for known risk factors. Reduced SVD was recognized as an early sign of systemic vascular damage, which was closely related to retinal vascular diseases and systemic cardiometabolic events. Previous epidemiologic studies have demonstrated that SUA changes over time conferred significant risk for incident hypertension, type 2 diabetes (T2D), and rapid decline in kidney function.^18^^,^^28^ A population-based study of healthy adults in Hong Kong demonstrated that a lower SVD was significantly associated with older age, male gender, and longer AL, while a lower DVD was significantly associated with longer AL and greater creatine.^10^ This study advanced previous studies by incorporating SUA in the association analysis. The findings indicated that the correlation between UA and cardiovascular risk may partly be explained by the alternation in the microvascular system by UA.

CC plays a key role in providing oxygen and nutrients to the outer retina. Alterations of CC have been implicated in age-related macular degeneration, diabetic retinopathy, pathologic myopia, and central serous chorioretinopathy. Furthermore, it was found that CFD predicted the outcomes in patients with posterior uveitis. In addition, CC abnormalities have been observed in systemic vascular disease and pregnancy-related complications. Therefore, the quantification of CC can provide important information about the pathogenesis of ocular and systemic diseases. A recent study has shown that CFD in OCTA is affected by age, blood pressure, and lipid levels, but SUA was not analyzed in the study.^11^ The present study found that high SUA led to increased CFD in both men and women after adjusting for other confounding factors. Therefore, SUA should be considered when interpreting CC changes in normal and diseased subjects.

The mechanism responsible for the association of a high level of SUA with impaired retinal capillary plexus and CC remains elusive for several possible reasons. First, elevated SUA can result in hypertension. In patients with untreated primary hypertension and adolescents with new-onset hypertension, the prevalence rates of elevated SUA were 25% to 60% and 90%, respectively.^29^ Second, chronic hyperuricemia leads to activation of the renin–angiotensin system, inhibition of nitric oxide synthetase, and renal vascular constriction, further resulting in the development of atherosclerosis and hypertension. Third, a high UA level induces endothelial dysfunction, which can be improved by allopurinol therapy. A hyperuricemic animal model has demonstrated that endothelial dysfunction can be caused by the impairment of nitric oxide synthesis.^30^ Fourth, SUA stimulates inflammation, oxidative stress, and cytokine secretion. After SUA-lowering treatment, the production of reactive oxygen species by endothelial cells increased by activating NADPH oxidase.^31^ Finally, SUA is associated with the proliferation of vascular smooth muscle cells, which is the crucial process of vascular remodeling and atherosclerosis.^32^ More studies are needed to clarify the underlying pathophysiology.

The influence of SUA on retinal microcirculation is more pronounced in females. Prior research on sex-dependent associations of SUA with diabetes mellitus and its complications has been documented.^22^^,^^33^^,^^34^ Although hyperuricemia poses an increased risk of developing diabetes mellitus in both sexes, females with hyperuricemia are more likely to develop diabetes than males with hyperuricemia.^35^ A Japanese study showed that higher SUA levels are associated with an increased risk of new-onset diabetic retinopathy in male patients but not in female patients.^22^^,^^33^ Other studies have reported that increased SUA is more harmful to women than to men.^34^^,^^36^^–^^38^ A study of a Chinese coastal population found that each standard deviation increase in SUA was independently associated with a 1.68-µm increase in retinal venous caliber in women but not significantly in men.^39^ This study noted that women's SVD measurements appeared to be more sensitive to elevated SUA levels than men's. More studies are needed to clarify the effect of gender on SUA and OCTA parameters.

Associations of SUA levels with cardiovascular diseases, diabetes mellitus, and its complications have long been described in the literature. A meta-analysis of a cohort study involving 958,410 participants revealed that coronary heart disease mortality increased by 13% for each 1-mg/dL UA elevation.^40^ Another meta-analysis of 230,000 participants demonstrated that hyperuricemia resulted in a 1.47 times risk for incident stroke and 1.26 times risk for stroke mortality after adjusting known risk factors.^41^ In patients with T2D, SUA level was found to be associated with worsening in severity of diabetic retinopathy and nephropathy over 3 years.^42^ Consistent with previous studies, this study demonstrated that the known cardiovascular risk factors increased in the hyperuricemia group, such as BMI, WHR, SBP, DBP, triglyceride, and total cholesterol, while protective factors decreased in the hyperuricemia group, such as HDL-C and serum creatine (Table 1). These results suggest that a high UA level increases the risk of cardiovascular disease in this population.

The strength of this study lies in the homogeneous population, standardized protocol, and state-of-the-art OCTA device. In addition, a variety of confounding variables, such as weight, height, and blood pressure, were measured by experienced technicians with standardized methods to minimize information bias. Our study, therefore, adds to the current knowledge of quantitative data on the normal retinal vascular system. Nevertheless, the limitations of this study should be noted. First, the UA data were obtained by one test, but UA concentration variations may exist during the year. However, it was not pragmatically feasible to perform multiple UA tests in this large population. Second, the cross-sectional analyses prevented the cause–effect inference. It would be more informative and persuasive if the baseline and longitudinal changes of SUA levels and OCTA parameters were available. Third, the details of the drug information among participants were not collected (e.g., angiotensin-converting enzyme inhibitor and statin). It has been reported that these drugs might influence the measurements of retinal vascular calibers.^43^^,^^44^ Fourth, the diurnal variation in the vessel density among hyperuricemia and normal participants cannot be assessed in the current study. Finally, the sample population was mainly formed by the Chinese Han population, which may introduce selection bias. Therefore, further studies are needed to confirm the findings of this study in other populations.

## Conclusion

In summary, a higher UA level was independently associated with reduced SVD and increased CFD in the study population. Women appear to be more sensitive to high SUA levels than men. These findings suggest that elevating UA concentration may play a role in the development and progression of cardiovascular diseases through microvascular alteration, as demonstrated by OCTA parameters. Further studies are needed to clarify the underlying pathophysiology and determine whether UA-targeted therapy improves chorioretinal microcirculation.

## Supplementary Material

Supplement 1
