# Supplementary material for: Retinal Microvasculature and Choriocapillaris Flow Deficit in Relation to Serum Uric Acid Using Swept-Source Optical Coherence Tomography Angiography
Source: Transl Vis Sci Technol. 2022 Aug 10;11(8):9. doi: 10.1167/tvst.11.8.9 (PMC9382346; doi:10.1167/tvst.11.8.9)
Supplement: Supplement 1 [file tvst-11-8-9_s001.docx]

**Supplemental Table 1.** Regional variations of retinal vasculature and choriocapillaris flow in ETDRS sectors between the subjects with normal SUA and hyperuricemia.

|  | **Normal SUA**  **(mean ± SD)** | **Hyperuricemia**  **(mean ± SD)** | **P-value**^†^ |
| --- | --- | --- | --- |
| **SVD, %** |  |  |  |
| Outer superior | 44.55±3.84 | 43.45±4.33 | <0.001 |
| Inner superior | 37.41±5.90 | 36.11±6.39 | 0.002 |
| Outer nasal | 42.42±4.17 | 41.92±4.28 | 0.094 |
| Inner nasal | 33.83±6.49 | 32.73±6.94 | 0.018 |
| Outer inferior | 41.76±4.33 | 41.13±5.34 | 0.056 |
| Inner inferior | 34.28±6.41 | 33.58±6.72 | 0.126 |
| Outer temporal | 38.40±5.08 | 36.88±5.90 | <0.001 |
| Inner temporal | 34.95±5.97 | 33.40±6.68 | <0.001 |
| **DVD, %** |  |  |  |
| Outer superior | 46.41±3.05 | 46.46±3.40 | 0.824 |
| Inner superior | 46.75±3.18 | 46.74±3.50 | 0.952 |
| Outer nasal | 45.80±3.13 | 46.03±3.10 | 0.279 |
| Inner nasal | 45.72±3.80 | 45.59±4.13 | 0.633 |
| Outer inferior | 44.69±3.44 | 44.94±3.72 | 0.319 |
| Inner inferior | 45.78±4.03 | 45.87±3.84 | 0.751 |
| Outer temporal | 46.84±2.71 | 46.72±3.21 | 0.529 |
| Inner temporal | 45.06±3.53 | 44.96±3.69 | 0.710 |
| **CFD, %** |  |  |  |
| Outer superior | 9.29±1.68 | 9.42±2.12 | 0.322 |
| Inner superior | 10.15±2.98 | 9.98±3.19 | 0.423 |
| Outer nasal | 10.05±2.05 | 10.43±1.93 | 0.008 |
| Inner nasal | 9.76±2.83 | 10.03±3.14 | 0.181 |
| Outer inferior | 8.98±1.74 | 9.27±2.11 | 0.026 |
| Inner inferior | 9.56±2.41 | 9.92±2.72 | 0.038 |
| Outer temporal | 8.96±1.78 | 9.43±2.24 | 0.001 |
| Inner temporal | 9.73±2.58 | 9.95±2.61 | 0.235 |

Abbreviations:SUA=serum uric acid; SVD=superficial vessel density; DVD=deep vessel density; CFD=choriocapillaris flow deficit; SD=standard deviation.

† Student’s t-test
